# DEVELOPING A MOBILE PHONE APPLICATION TO DETECT SOCIAL ISOLATION OVER MULTIPLE TIME POINTS

**DOI:** 10.1093/geroni/igac059.2610

**Published:** 2022-12-20

**Authors:** Rajlaxmi Biyani, Anupam Joshi, Gretchen Tucker, Dana Bradley, Carmen Sceppa

**Affiliations:** University of Maryland Baltimore County, Fairfax, Virginia, United States; University of Maryland Baltimore County, Baltimore, Maryland, United States; University of Maryland, Baltimore, Columbia, Maryland, United States; University of Maryland, Baltimore County, Baltimore, Maryland, United States; Northeastern University, Boston, Massachusetts, United States

## Abstract

Social isolation is an important predictor of mortality and morbidity in older persons. The increase in social isolation is often a result of major life events such as retirement, leading to the loss of social connections. The effects of the pandemic have exacerbated the loss of social connectedness. Measures to ensure social distancing have made technologies like voice and video calling, text messaging, and other phone mediated communication important resources for connecting people. Measuring social isolation often requires periodic administration of a paper survey instrument, like the Lubben Social Network Scale (LSNS). The approach was taken by Lubben for predicting a person’s social isolation level typically involves looking at their general frequency of interactions with family and friends along with the specific frequencies of private, tentative discussions happening during those interactions. This paper explores the process used to develop a tool to measure social isolation continuously and automatically by using an older person’s frequency and tone of telephonic conversations. We categorized a person’s family and friends into three sub-categories based on the self-reported level of closeness shared with them. We then analyzed the person’s conversation (content and tones). Lastly, we trained the deep learning model (Watson) using these captured tone values to determine the social isolation index of the individual. Our preliminary results indicate that we are able to correctly predict their degree of social isolation as judged by Lubben’s scale using their phone conversations. This technology may be promising for the assessment and intervention of social isolation.

